# Therapeutic effect of Xuebijing combined with thymosin on hemorrhagic fever with renal syndrome: A protocol of systematic review and meta-analysis

**DOI:** 10.1097/MD.0000000000020262

**Published:** 2020-05-15

**Authors:** Chun-mei Li, Qi Sun

**Affiliations:** a Department of Infectious Diseases, First Affiliated Hospital of Jiamusi University, Jiamusi, 154002; b Department of Infectious Diseases, Weihai Central Hospital, Weihai, 264400, China.

**Keywords:** effect, hemorrhagic fever, randomized controlled trial, renal syndrome, safety, thymosin, Xuebijing

## Abstract

**Background:**

The goal of this study is to assess the therapeutic effect of Xuebijing combined with thymosin (XBJ-T) for the treatment of patients with hemorrhagic fever with renal syndrome (HFRS).

**Methods:**

We will search the electronic databases of Cochrane Library, PUBMED, EMBASE, PsycINFO, Scopus, Opengrey, Cumulative Index to Nursing and Allied Health Literature, Web of Science, Google scholar, Allied and Complementary Medicine Database, and Chinese Biomedical Literature Database from inception to the present. No language and publication status will be employed in this study. Based on the predefined eligibility criteria, selection of study and data extraction will be performed by 2 researchers independently. Study quality will be assessed using Cochrane risk of bias tool. We will apply RevMan 5.3 software to pool and analyze the extracted data.

**Results:**

This study will assess the therapeutic effect of XBJ-T for the treatment of patients with HFRS.

**Conclusion:**

The findings of this study may provide systematic evidence to judge whether XBJ-T is an effective and safety intervention for HFRS.

**Study registration number:**

INPLASY202040068.

## 1 Introduction

Hemorrhagic fever with renal syndrome (HFRS) is a severe life threatening disease due to the hantavirus.^[1,2,3]^ It is often carried and transmitted by rodents.^[4,5,6,7]^ It has been reported that hantavirus infection affects 30,000 individuals annually,^[8]^ and about 90% of the HFRS cases occur in Asian countries, especially in China, Japan, and South Korea.^[9,10,11,12,13,14]^ Fortunately, previous studies have reported that Xuebijing combined thymosin (XBJ-T) can effectively treat patients with HFRS.^[15,16,17,18,19,20]^ However, there is not systematic review and meta-analysis that has assessed the therapeutic effect of XBJ-T for the treatment of patients with HFRS. Therefore, this study will appraise the effect of XBJ-T for the treatment of patients with HFRS.

## 2 Methods and analysis

### 2.1 Study registration

This study protocol has reported following the standards of the Preferred Reporting Items for Systematic Review and Meta-Analysis Protocols Statement,^[21]^ and is registered on INPLASY202040068.

### 2.2 Ethics and dissemination

This study will not need ethical approval, because it will not obtain individual patient data. The results of this study will be published through on a peer-reviewed journal.

### 2.3 Eligibility criteria

#### 2.3.1 Types of studies

This study will include randomized controlled trials (RCTs) on assessing the effect of XBJ-T for the treatment of patients with HFRS. We will exclude other studies, such as case report, case series, and reviews.

#### 2.3.2 Types of participants

All participants (18 years old or older) who clinically diagnosed as HFRS will be included in this study. No race, sex, and educational background will be applied.

#### 2.3.3 Types of interventions

In the intervention group, all forms of XBJ-T regardless of dosage, treatment period will all be included in this study.

In the control group, we will consider any management as their comparators, but not XBJ-T.

#### 2.3.4 Types of outcomes

The primary outcomes are positive rate of specific IgM and positive rate of specific IgG. They were measured by colloidal gold immuno-dot assay or others.

The secondary outcomes are rate of mortality, improving viremia, clinical conditions, restoring kidney function and platelet account, and occurrence rate of complications.

### 2.4 Search strategy

This study will search the electronic databases of Cochrane Library, PUBMED, EMBASE, PsycINFO, Scopus, Opengrey, Cumulative Index to Nursing and Allied Health Literature, Web of Science, Google scholar, Allied and Complementary Medicine Database, and Chinese Biomedical Literature Database from the beginning of each database to the present. This study will not employ any language and publication status limitations. The example of Cochrane Library is built (Table 1). We will also adapt similar search strategies to other electronic databases.

**Table 1:**
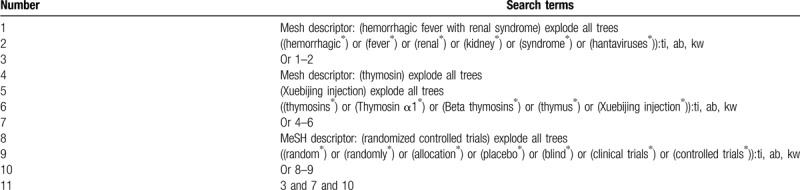
Search strategy applied in Cochrane Library.

In addition to the electronic databases, we will also manually search other related journals and conference proceedings, as well as the reference lists of relevant reviews.

### 2.5 Study selection

**Figure 1:**
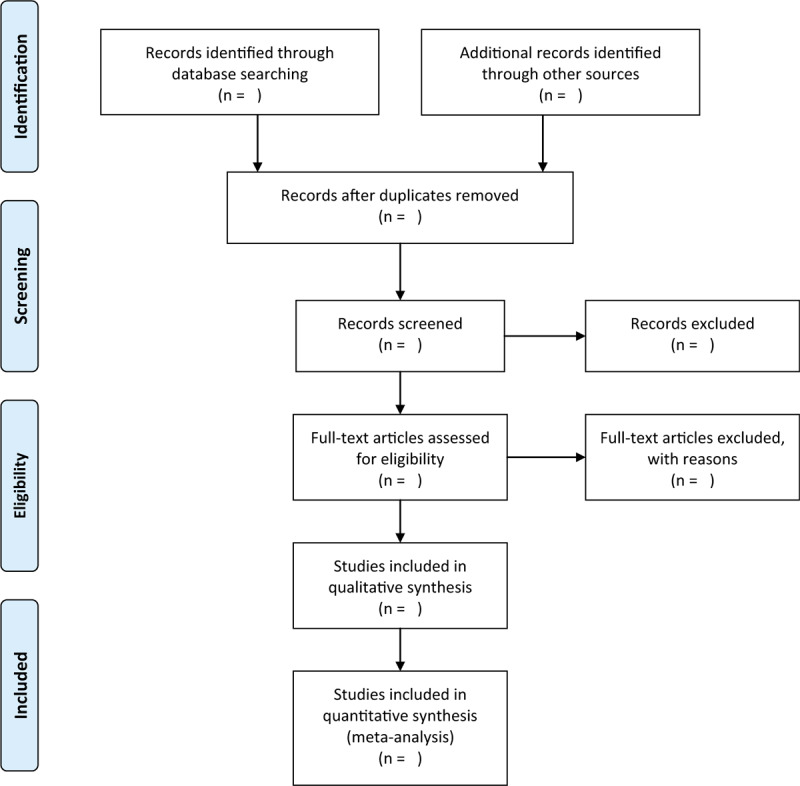
Flowchart of study selection.

Two researchers will independently read the topics and abstracts to identify the possible records that might be included. All duplicates and irrelevant studies will be removed. After that, we will read full-text of the remaining studies to further determine if they meet all inclusion criteria. Inconsistencies between 2 researchers will be solved with the help of a third researcher by discussion. The study selection process will be demonstrated in a flowchart (Fig. 1).

### 2.6 Data extraction and management

Two researchers will independently utilize the pre-designed data extraction sheet to collect the data. It includes data from several aspects, including general study information, patient characteristics, study setting, study methods, sample size, interventions, comparators, outcomes, and other relevant indicators. If there are any inconsistencies between 2 researchers, a third researcher will help to resolve them by discussion. If we identify any missing data or unclear data, we will contact primary authors to request this information by email.

### 2.7 Risk of bias assessment

Two researchers will independently assess the risk of bias for each included trial using Cochrane risk of bias tool. It covers 7 aspects and each one is determined as 3 levels: low, unclear, and high risk of bias. Any inconsistencies will be resolved by a third researcher through consultation.

### 2.8 Data synthesis and analysis

RevMan 5.3 software will be used to conduct data synthesis and data analysis. As for continuous outcome indicators, they will be expressed as mean difference and 95% confidence intervals (CIs). As for dichotomous outcome indicators, they will be exerted as risk ratio and 95% CIs. We will check statistical heterogeneity across included trials using *I*^2^ test. *I*^2^ ≤ 50% indicates low heterogeneity, while *I*^2^ > 50% means obvious heterogeneity. If the trials are sufficiently similar, we will pool the data from original studies using a fixed-effect model when *I*^2^ ≤ 50%. On the other hand, if *I*^2^ > 50%, we will synthesize the data using a random-effect model. We will also carry out subgroup analysis to explore the causes of obvious heterogeneity.

### 2.9 Additional analysis

#### 2.9.1 Subgroup analysis

We will undertake subgroup analysis to identify the source of heterogeneity based on the different interventions, comparators, and outcome indicators.

#### 2.9.2 Sensitivity analysis

We will perform sensitivity analysis to examine the robustness of pooled results by taking away low quality studies.

#### 2.9.3 Reporting bias

When at least 10 trials are included, we will conduct funnel plots and Egger's regression test to investigate if there are any reporting biases.^[22,23]^

## 3 Discussion

This systematic review is the first study to evaluate the therapeutic effect of XBJ-T for the treatment of patients with HFRS. It will comprehensively search literatures sources, including electronic databases and grey literatures to avoid missing any potential studies. Two researchers will independently carry out study selection, data extraction, and study quality evaluation. If we examine any conflicts, we will invite a third researcher to solve any divergences between 2 researchers. The results of this study will provide latest evidence on the therapeutic effect of XBJ-T for the treatment of patients with HFRS. Its findings may provide evidence for clinical practice and future studies.

## Author contributions

**Conceptualization:** Chun-mei Li, Qi Sun.

**Data curation:** Chun-mei Li, Qi Sun.

**Formal analysis:** Chun-mei Li, Qi Sun.

**Funding acquisition:** Chun-mei Li.

**Investigation:** Chun-mei Li.

**Methodology:** Qi Sun.

**Project administration:** Chun-mei Li.

**Resources:** Qi Sun.

**Software:** Qi Sun.

**Supervision:** Chun-mei Li.

**Validation:** Chun-mei Li, Qi Sun.

**Visualization:** Chun-mei Li, Qi Sun.

**Writing – original draft:** Chun-mei Li, Qi Sun.

**Writing – review & editing:** Chun-mei Li, Qi Sun.
